# Map-based cloning of the APRR2 gene controlling green stigma in bitter gourd (*Momordica charantia*)

**DOI:** 10.3389/fpls.2023.1128926

**Published:** 2023-05-10

**Authors:** Jinyi Zhan, Jian Zhong, Jiaowen Cheng, Yuhui Wang, Kailin Hu

**Affiliations:** ^1^ Key Laboratory of Biology and Genetic Improvement of Horticultural Crops (South China), Ministry of Agriculture and Rural Affairs/Guangdong Vegetables Engineering Research Center, College of Horticulture, South China Agricultural University, Guangzhou, China; ^2^ State Key Laboratory of Crop Genetics & Germplasm Enhancement and Utilization, College of Horticulture, Nanjing Agricultural University, Nanjing, China

**Keywords:** bitter gourd, stigma color, BSA-seq, McAPRR2, fine mapping

## Abstract

Bitter gourd is an economically important vegetable and medicinal crop distinguished by its bitter fruits. Its stigma color is widely used to assess the distinctiveness, uniformity, and stability of bitter gourd varieties. However, limited researches have been dedicated to genetic basis of its stigma color. In this study, we employed bulked segregant analysis (BSA) sequencing to identify a single dominant locus *McSTC1* located on pseudochromosome 6 through genetic mapping of an F_2_ population (*n* =241) derived from the cross between green and yellow stigma parental lines. An F_2_-derived F_3_ segregation population (*n* = 847) was further adopted for fine mapping, which delimited the *McSTC1* locus to a 13.87 kb region containing one predicted gene *McAPRR2* (*Mc06g1638*), a homolog of the *Arabidopsis* two-component response regulator-like gene *AtAPRR2*. Sequence alignment analysis of *McAPRR2* revealed that a 15 bp insertion at exon 9 results in a truncated GLK domain of its encoded protein, which existed in 19 bitter gourd varieties with yellow stigma. A genome-wide synteny search of the bitter gourd *McAPRR2* genes in Cucurbitaceae family revealed its close relationship with other cucurbits APRR2 genes that are corresponding to white or light green fruit skin. Our findings provide insights into the molecular marker-assisted breeding of bitter gourd stigma color and the mechanism of gene regulation for stigma color.

## 1 Introduction

Flower color, especially petal color, is the most noticeable variation of floral features in angiosperms with colors spanning the whole color spectrum perceived by human and pollinators. There is general agreement that the diversity of flower colors in angiosperms is mostly determined by variation in the interactions with pollinators *via* natural selection, in part because pollinators tend to enhance assortative mating (Gervasi and Schiestl, 2017; Trunschke et al., 2021). In addition to the most visible petal color, other types of flower colors include pollen, gynoecium and inflorescence colors polymorphisms (Carlson and Holsinger, 2010; Koski et al., 2020). The color of stigma may also differ across plant varieties and be distinguishable from the petal background to attract pollinators, such as tulip, melon, and sunflower, which may further enhance the crop production (Miklič et al., 2002; Miller et al., 2011; Lv et al., 2022b). In another instance, the stigma of pistachio (*Pistacia vera* L.) bears exclusive colors at various developmental stages, which has been utilized as an indicator for the optimum time of artificial pollination (Sharifkhah et al., 2020). Botanically, stigma color is an essential morphological characteristic for differentiating the species or types of plants. The green stigma, for instance, is one of the phenotypic traits used to differentiate the prickly pear cactus *Opuntia Bonaerensis* from its close relative *Opuntia elata* (Köhler et al., 2020).

At present, few research have reported the genetic basis of stigma color. Studies on pecan (*Carya illinoinensis*) and tomato (*Solanum lycopersicum*) suggest that the green stigma is regulated by a single dominant gene with their green stigma dominating over red and yellow stigma, respectively (Beedanagari et al., 2005; Zhao et al., 2017). In contrast, studies for stigma color for melon (*Cucumis melo*) and rice (*Oryza sativa*) propose polygenic model (Meng et al., 2021; Qiao et al., 2021; Lv et al., 2022b). In melon, two quantitative trait loci (QTL) *SC2.1* and *SC8.1* on chromosomes (Chr) 2 and Chr 8, have been mapped for regulating the green stigma, however their underlying causative genes are remain unclear (Qiao et al., 2021; Lv et al., 2022b). In a rice *indica* cultivar, its purple apiculi and stigma were genetic regulated by the interaction of two genes involved the anthocyanin biosynthesis pathway: *OsC1* (encoding a R2R3-MYB transcription factor) and *OsDFR* (encoding a dihyroflavonol 4-reductase) (Meng et al., 2021). Recent two transcriptomic studies aimed at revealing the gene network were conducted and showed that the yellow stigma in tomato is very likely due to the accumulation of yellow-colored flavonoid naringenin chalcone, whereas the melon yellow stigma is due to break-down of chloroplast structure interfering chlorophyll biosynthesis (Zhang et al., 2017; Lv et al., 2022a). Despite the crucial functional roles of stigmas play in plant reproduction, there has not been sufficient researches dedicated to the color of stigma.

Bitter gourd (syn. bitter melon; *Momordica charantia* L.; 2x=2n=22), belongs to the Cucurbitaceae family and the *Momordica* genus, is an economically important vegetable and medicinal crop marked by its bitter fruits. Bitter gourd is abundantly cultivated in the tropical and subtropical countries or regions, including China, India, Malaysia, Africa, and South America. As the next-generation long-read sequencing method has recently been applied to bitter gourd, the high-quality whole genome assemblies are now available for both bitter gourd cultivars (OHB3-1 and Dali-11) and wild accession (TR), which greatly facilitate the process of addressing fundamental issues and advancing bitter gourd breeding (Urasaki et al., 2017; Cui et al., 2020; Matsumura et al., 2020). In addition, it provides intriguing potential for re-investigating those previously documented horticultural traits. For example, the underlying gene of the black seed coat color of bitter gourd was pinpointed to a 13.2 kb region containing only one candidate gene, *MC03g0810*, encoding a polyphenol oxidase (PPO) (Kole et al., 2012; Zhong et al., 2022). Other quantitative traits, such as fruit size and yield, were also thoroughly investigated by applying high-density genetic map (Rao et al., 2021).

Similar to other cucurbits, bitter gourd is monoecious with male and female flowers produced on the same plant. In female flowers, the stigmas often present two colors: yellow and green. This characteristic is widely used to assess the distinctiveness, uniformity, and stability of a bitter gourd variety (UPOV: TG/235/1,Guidelines for the conduct of tests for distinctness,uniformity and stability—Bitter gourd, NEQ). The green stigma is easily distinguished from the background of yellow petals of bitter gourd which is suggested be more attractive to the pollinators (such as bees and flies) (Miller et al., 2011; Lv et al., 2022b). The first genetic study for stigma color of bitter gourd was conducted used an F_2_ population derived from a cross between “Taiwan White” and “CBM12” which had distinct fruit, stigma, and seed colors (Kole et al., 2012). According to Kole et al. (2012), stigma color is regulated by a single dominant locus that is independently inherited and was not found to be related with other fruit traits (e.g., fruit color, fruit surface structure, seed color) (Kole et al., 2012). However, the fine mapping and cloning of the stigma color gene in bitter gourd have not yet been accomplished.

In the present study, we developed a mapping population with 241 F_2_ plants from the cross of two bitter gourd inbred lines S051 (green stigma) and S093 (yellow stigma), and further applied the bulked-segregant analysis sequencing (BSA-seq) method to identify the inheritance locus/loci. Through map-based cloning using an 847 F_3_ population, we identified that *McAPRR2* as the best candidate gene for stigma color. We further developed a molecular marker linked to the variation presented in *McAPRR2*. The genome-wide survey of *APRR2* genes in Cucurbitaceae revealed that they undergo whole genome duplications (WGDs) event and may have redundant or specific functions in stigma color modulation. This work will allow for the application of marker-assisted selection (MAS) for bitter gourd breeding and further increase our understanding of the molecular mechanism underlying stigma color development.

## 2 Materials and methods

### 2.1 Plant materials and phenotyping

Two bitter gourd inbred lines, S051 with green stigma and S093 with yellow stigma, were the main subjects in the present study. They were used to generate F_2_ and F_2_-derived F_3_ populations for genetic mapping of stigma color. The F_2_-derived F_3_ population was constructed from self-pollination of F_2_ individuals exhibiting heterozygous genotype in preliminary mapping interval. A total of 241 F_2_ individuals and 847 F_3_ individuals were subjected to preliminary mapping using bulk-segregant-analysis (BSA)-seq and fine-mapping for the candidate genes, respectively. All plant materials were grown at QiLin north Campus Teaching & Research Base of South China Agricultural University, Guangzhou, China (23°N, 113°E). The stigma color from mature female flowers was classified using color card of RAL D9 DESIGN CYMPHONY OF COLOURS. The stigma colors close to RAL 110-80-70, 120-70-75, and 100-60-60 were assigned to green, while those are comparable to RAL 100-80-70, 95-80-70, and 90-80-90 were defined as yellow (**Figure S1A**).

### 2.2 Bulked-segregant analysis

BSA was employed for rapid identification of the stigma color locus. Two DNA bulks, the green stigma and yellow stigma DNA pools, were constructed each mixing equal amounts of genomic DNA from 30 individuals with green and yellow stigmas from F_2_ population. The two DNA pools were subjected to whole genome resequencing with Illumina GAIIx sequencer (Takagi et al., 2013). Raw reads were filtered by SOAPnuke software for removing adapters contamination and low-quality reads and further filtered by SAMtools with ‘view-q30’ standard parameter for sorting and quality control (Li et al., 2009; Chen et al., 2018). The Variants Calling followed the BWA-GATK workflow using the bitter gourd Dali-11 v1 as reference genome (Li and Durbin, 2009; Cui et al., 2020). The following filtration standards high quality Single Nucleotide Polymorphism (SNP) were applied: Genotype Quality >= 50, removing heterozygous locus between parents and SNP locus lacked in green and yellow pools (McKenna et al., 2010).

The filtered SNPs and insertion-deletion (InDel) were adopted for SNP-index and Euclidean distance (ED) algorithm analysis for BSA (Abe et al., 2012; Hill et al., 2013). The formulas of SNP-index and the Δ(SNP-index) were used as follow: *SNP-index = AD_r_/(AD_d_+AD_r_)*, *Δ(SNP-index) = SNP-index (yellow pool)-SNP-index (green pool)*, where *AD_r_* and *AD_d_* represent yellow and green stigma allele depth, respectively. An average SNP-index and Δ(SNP-index) of each position were calculated *via* 200 kb sliding window with 100 kb step, windows out of 95% and 99% confidence intervals of the Δ(SNP-index) were treated as significant and extremely significant windows respectively (Abe et al., 2012). The formula of ED^2^ was used as follow: *ED^2^ = (A_yellow pool_-A_green pool_)^2^+(C_yellow pool_-C_green pool_)^2^+(G_yellow pool_-G_green pool_)^2^+(T_yellow pool_-T_green pool_)^2^*, where A, C, G, T represent the proportion of mutant type and reads respectively (Hill et al., 2013).

### 2.3 Linkage analysis and fine mapping

The polymorphic variants (SNPs and InDels) between the two parental lines S051 and S093 were further developed as molecular markers using Primer 3 web (https://primer3.ut.ee/) and SnapGene software for Cleaved Amplified Polymorphic Sequences (CAPS). These markers were applied to genotype 241 F_2_ individuals to construct a linkage map for the candidate region obtained from BSA-seq analysis *via* JoinMap 4 software. For further narrow down the candidate region, two flanking markers ST5 and ST8 were used to genotype additional 847 F_3_ individuals to identify recombinants plants. Additional markers were explored in the region defined by flanking markers. Information of all markers and primers used in this study is provided in **Table S1**.

### 2.4 DNA annotation, gene predication, and full-length cDNA cloning

The candidate genes in the 13.87 kb region of Dali-11 v1, OHB3-1v2, and TR v1 reference genome, and the predicted genes by the FGENESH program (http://www.softberry.com/berry.phtml) were compared and contrast. We then designed three pairs of primers to obtain the full-length CDS of *McAPRR2* in both S051 and S093 accordingly (**Table S1**). The conserved domains were annotated with NCBI Conserved Domain Search (https://www.ncbi.nlm.nih.gov/Structure/cdd).

### 2.5 Gene expression analysis

The expression dynamics of *McAPRR2* were examined in different stages of the stigma from the two parental lines, which included 1 to 4 days before flowering (DBF) and the flowering day (FD). The tissue-specific expression of *McAPRR2* was examined in the following organs as well: the root, stem, and leaf tissues were collected from 30 days seedlings; the stamen and petals of both male and female flowers were collected at FD; the ovary tissues were harvested from 4DBF to FD, as well as mature fruit skin tissue. All of the tissue samples were collected with three biological replicates, which were frozen temporarily in liquid nitrogen, and then stored at −80 °C for RNA isolation. The reverse transcription was performed using total RNA extracted by Eastep^®^ Super Total RNA Extraction Kit (Promega, Shanghai) as follows: 500 ng RNA template, 2 μL Eastep^®^ RT Master Mix (5×) and additional nuclease-free water to 10 μL reaction system. The qPCR procedure followed the manufacturer’s instruction of Eastep^®^ qPCR Master Mix with the bitter gourd endogenous actin gene *Mc01g0724*. The fold changes in expression level of stigma and other tissues were relative subjected to stigma at 4DBF of S093 and stigma at FD of S093 using 2^-ΔΔCT^, respectively.

### 2.6 Phylogenetic analysis

The genome-wide syntenic search using *McAPRR2* as probe sequence were performed in the synteny module of CuGenDBv2 (http://cucurbitgenomics.org/v2/); their protein of *APRR2* in Cucurbitaceae family were downloaded from CuGenDBv2 as well (Yu et al., 2022). The *APRR2* genes from apple (*LOC103440803*), tomato (*LOC101245957*), pepper (*CA06g13040*, *CA00g25180*/*Capana01g000809*), eggplant (*Sme2.5_00446.1_g00003.1*) and Arabidopsis (*AT4G18020*) were downloaded from NCBI or genome database for tree construction as well (Hwang and Sheen, 2001; Jeong et al., 2020; Lim et al., 2021; Fang et al., 2023). The Arabidopsis *AtAPRR1* (*AT5G61380*), *AtGLK1* (*AT2G20570*), and *AtGLK2* (*AT5G44190*) were included for outgroup (Hwang and Sheen, 2001; Susila et al., 2023).

Sequence alignment were performed using DNAMAN V6 software. Bootstrap method with 1000 replications and Poisson model were used as phylogeny test and substitution model, respectively, while constructing the neighbor-joining tree in MEGA 11 software (Tamura et al., 2021). The tree was visualized with R/ggtree package (Yu et al., 2017). The conserved motifs of APRR2 proteins were evaluated *via* online MEME software (http://meme-suite.org/). The positions of the conserved domains were visualized in R/ggplot2 package.

## 3 Result

### 3.1 Inheritance of the stigma color

**Figure 1:**
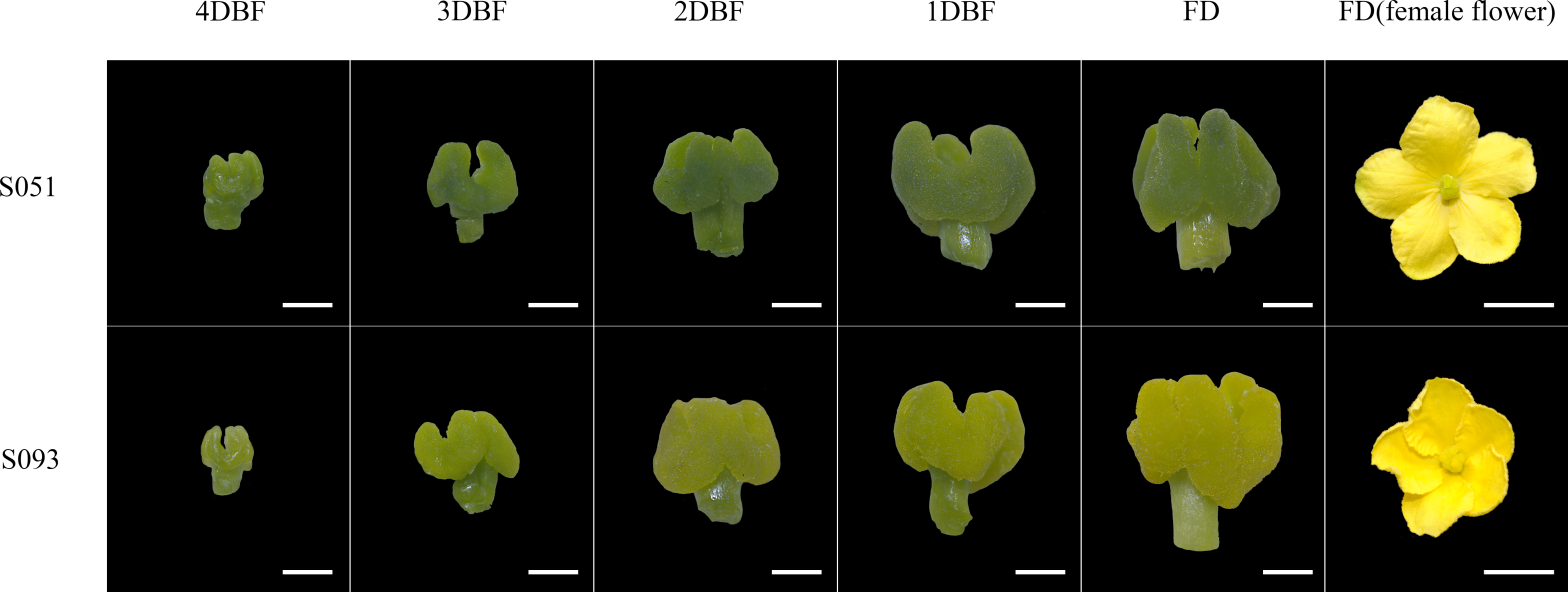
Phenotype of stigma color in two parental lines (S051 and S093) from four days before flowering (4DBF) to flowering day (FD). Bars in stigma and female flower represent 1 mm and 1 cm, respectively.

To reveal the genetic inheritance of the stigma colors of bitter gourd, we employed two bitter gourd lines, S051 and S093, of which stigma displayed distinctive green and yellow colors that were distinguishable as early as four days before flowering (DBF) (**Figure 1**). Then, they were crossed to develop an F_2_ population consisting of 241 individuals for genetic study. To prevent any ambiguity in color assessment, we scored the stigma color of F_1_ plants and the F_2_ populations using a RAL D9 color card that differentiated a range of green and yellow hues through a series of Hue-Saturation-Lightness (HSL) values (**Figure S1**). According to the color card, the F1 individuals exhibited a green stigma phenotype implying that the green color of stigma is dominant over yellow stigma (**Table S1B**). Of the 241 F_2_ population, 191 plants have green stigma and 50 have yellow stigma, which is consistent with the 3:1 expected segregation ratio (*P*=0.13 in *χ^2^* test). It demonstrates that the green stigma in bitter gourd is regulated by a single dominant locus, which is designated as *McSTC1* (*Momordica charantia Stigma Color 1*) hereinafter.

### 3.2 Identifying the *McSTC1* locus based on BSA-seq

**Figure 2:**
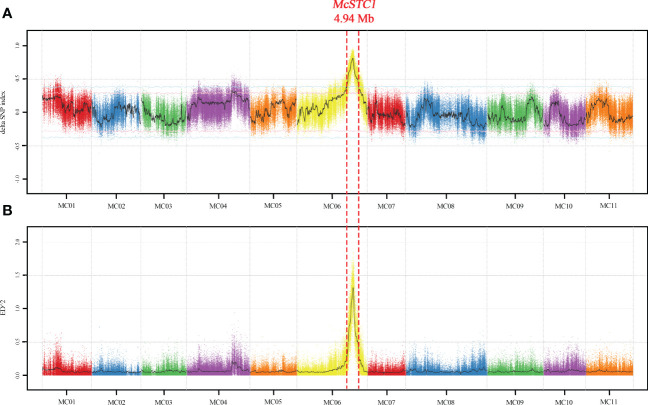
Bulked segregant analysis (BSA) of the *McSTC1* based on SNP-index and ED algorithm. **(A)** The Manhattan plot of Δ(SNP-index). The black line is the fitted Δ(SNP-index). The pink and blue lines represent the thresholds of top 5% and 1% respectively. **(B)** The Manhattan plot of ED^2^ value. The confidence interval of *McSTC1* is limited with red dotted lines in 4.94-Mb at pseudochromosome 6.

To primarily determine the location of *McSTC1*, we conducted BSA-seq. Thirty plants with distinctly green and yellow stigma were selected from the 241 F_2_ population, and their DNA were pooled to create two DNA bulks. The two parental lines together with the two DNA bulks were subjected to whole genome resequencing. A total of average 16.63 Gb clean reads were obtained and the average read depth coverage is 53.83×. After proper filtering, an average of 97.08% reads at Q20 value were mapped to the reference Dali-11 v1 reference genome (Cui et al., 2020; http://cucurbitgenomics.org/v2/) (**Table S2**). Based on the uniquely mapped reads and proper filtering through GATK pipeline, a total of 392,662 SNPs were identified across 11 chromosomes between the green and yellow pools. These SNPs were adopted to calculate the Δ(SNP-index) and ED^2^ to determine the associated region with the stigma colors. By plotting against their positions along each pseudochromosome of the Dali-11 v1, a single significant peak with Δ(SNP-index) and ED^2^ ≥ 0.5 were identified on pseudochromosome 6 at a confidence level higher than 0.99 (**Figure 2**). The peak covered a physical interval of 4.94 Mb from 21.41 to 26.35 Mb, suggesting this candidate genomic region is the *McSTC1* locus and harbors the causative mutation (**Table S3**).

### 3.3 Linkage analysis and fine mapping of the *McSTC1* locus

**Figure 3:**
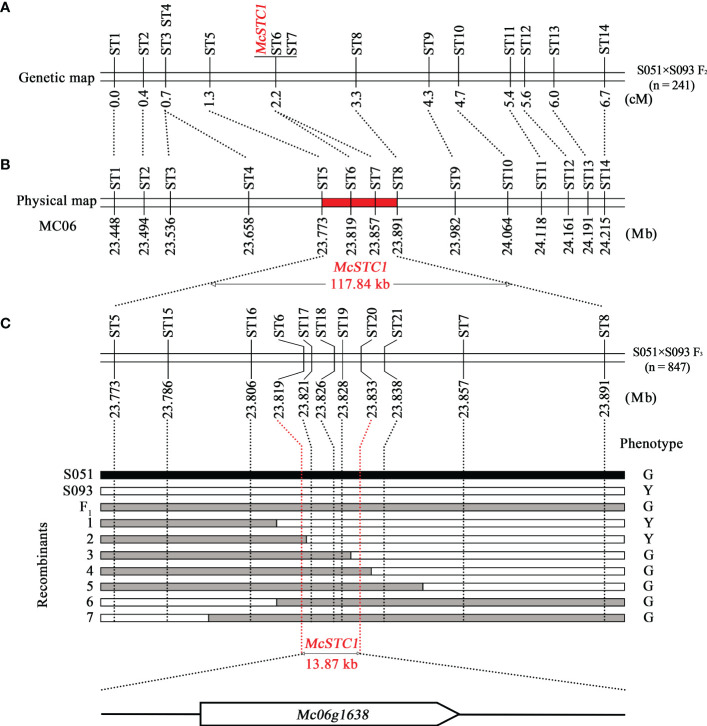
Linkage mapping and fine mapping of the *McSTC1 locus*. **(A)** The linkage map developed using 241 F_2_ population from the cross between S051 and S093. **(B)** The physical position of each marker according to reference genome Dali-11 v1.0. **(C)** The fine-mapping of *McSTC1* locus based on the recombinants identified from an F_3_ population consisting of 847 individuals. The black, white, and grey bar indicate the genotypes of S051 homozygotes, S093 homozygotes, and heterozygotes, respectively. The phenotype of G and Y are abbreviations of stigma color Green and Yellow, respectively.

In order to narrow down the interval of the *McSTC1* locus, regional linkage analysis and fine mapping were performed using the called variants obtained from BSA-seq. Based on the mapped reads of the two parental lines and two DNA pools, a total of 3,918 InDels and SNPs were identified in the 4.94 Mb candidate region (**Table S3**). Among them, we selected 17 InDel variants for polymorphic markers development, approximately evenly dispersed based on their physical positions from 22.67 to 25.41 Mb (**Table S1**). These markers were first applied to confirm in each F_2_ individual from two DNA pools. Accordingly, 14 markers (ST1-ST14, spanning from 23.45 to 24.22 Mb) that are closely linked to phenotype in two DNA pools were further utilized to genotype the primary 241 F_2_ population and to construct a regional linkage map covering a genetic distance of 6.7 cM (**Figure 3A**). The genetic mapping results showed that two markers, ST6 and ST7, were co-segregated with *McSTC1*. It then delimited *McSTC1* locus into a 117.84 kb region by the two flanking markers, ST5 and ST8 (**Figure 3B**).

To further pinpoint the candidate gene, we employed additional 5 InDel and 2 CAPS markers (ST15-ST21) in this region. Furthermore, a number of heterozygous F_2_ plants based on the heterozygous genotypes of ST1 and ST14 were self-pollinated to generate a fine-mapping F_3_ population. The two flanking markers (ST5 and ST8) were used to genotype a total of 847 F_3_ seedlings which identified 7 recombinants. Their stigma colors were then investigated in adult stage. By inspection of the genotypic and phenotypic data of the recombinants, the *McSTC1* is finally narrowed down into a 13.87 kb region defined by markers ST6 and ST20 (**Figure 3C**). Within this region, only one protein coded gene was annotated from the Dali-11 v1, namely *Mc06g1638* (*McAPRR2*), which encoded a homolog of *Arabidopsis* two-component response regulator-like protein (*APRR2*).

### 3.4 Sequence analyses of *Mc06g1638*

**Figure 4:**
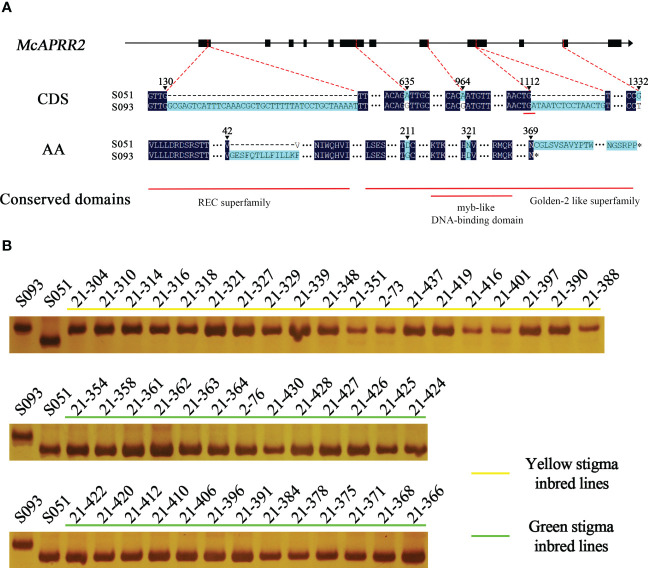
The sequence variance of *McAPRR2* and genotypic results of the 15 bp InDel variance of *McAPRR2* in 45 inbred lines of bitter gourd. **(A)** The gene structure, sequence and amino acid variance, and conserved domain analysis of *McAPRR2*. **(B)** A total of 19 lines had yellow stigma shared same genotype as S093 with the 15bp insertion. The other 26 lines showed green stigma are same as S051.

Currently, three reference genomes for bitter gourd are available: Dali-11 v1, OHB3-1 v2, and TR v1 (https://cucurbitgenomics.org/v2). All of the three reference genomes and FGENESH annotated one single *Mc06g1638* gene in 13.87 kb region, but they show discrepant prediction for its start codon and exon-intron structures. Thus, we designed three pairs of primers to further confirm the full-length cDNA sequences of *McAPRR2* in S051 and S093 (**Table S1**). We showed that the coding sequence of *McAPRR2* were 1560 bp and 1152 bp from S051 and S093 that are inconsistent with all reference genomes (CuGenDBv2: *Mc06g1638*, *Moc06g35570* and *MS005431.1*) (**Figure S2**). A total of five variants (3 SNPs and 2 InDels) were identified between the two parental lines: a synonymous SNP.G23827556T at the 11^th^ exon; two non-synonymous SNP.A23824615G and SNP.A23825627G at the 6^th^ exon and the 8^th^ exon resulted in D211G and N321D amino acid substitutions, respectively; two insertions in S093 with 39 bp and 15 bp at the 1^st^ exon and the 9^th^ exon resulted in an insertion of 13 amino acid and an introduction of premature stop codon, respectively (Genbank accession numbers OP972606 and OP972607 **Figure 4A** **;** **Figures S2, S3**).

The candidate gene *McAPRR2* of S051 allele encoded a complete protein of 519 amino acids with three conserved domains including phosphoacceptor receiver (REC) domain superfamily (cl19078) and Golden-2 like superfamily (GLK2, PLN03162) that nested with myb-like DNA-binding domain (myb SHAQKYF). The 39 bp InDel and the two non-synonymous SNPs located at REC domain and GLK2 domain, respectively, whereas the 15 bp InDel leads to a truncated GLK2 domain (**Figure 4A**).

We noticed that the bitter gourd cultivar Dali-11 has yellow stigma sharing the same variants with S093 including SNP.A23824615G, SNP.A23825627G, and present of the 15 bp insertion, while the wild bitter gourd TR has the green stigma with same variants as S051 (Cui et al., 2020). To confirm the association of these variants with stigma color, we phenotyped and genotyped 45 inbred lines which mainly collected in Southern Asia countries (**Table S4**). As a result, the 19 inbred lines with yellow stigma have the 15 bp insertion, and 26 inbred lines with green stigma lack it, which were consistent with genotyping results in S051 and S093 (**Figure 4B**; **Table S6**). To examine the non-synonymous SNP variants in the 45 lines, we designed a pair of primers covering the two SNPs to amplify the DNA of 45 inbred lines (**Table S1**). The Sanger sequencing result showed that the SNP.A23825627G is completely consistent with the phenotype while SNP.A23824615G is a bit deviated from it (**Table S6**). The SNP and InDel markers could be potentially applied for marker-assisted selection (MAS) for stigma color breeding in bitter gourd.

### 3.5 Developmental and tissue-specific expression analysis of *McAPRR2*

**Figure 5:**
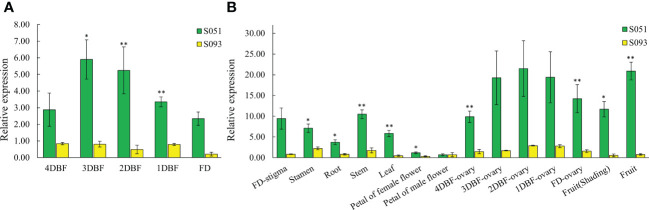
The relative expression between the parental lines S051 and S093. **(A)** The relative developmental expression level of stigma tissues from 4 days before flowering (DBF) to flowering day (FD). **(B)** The relative tissue-specific expression level in other organ tissues including stamen, petals, root, stem, leaf, ovary from 4DBF to FD, and fruit skin. The data are presented as the average values of three biological replicates (Mean ± SE). * and ** indicate the statistically significant differences of expression level at *P* < 0.05 and *P <*0.01 level, respectively, according to *student t-test*.

In order to address the expression pattern of *McAPRR2*, we collected five developmental stages of stigma from 4 DBF to the date it blossoms and other organ tissues including stamen, petal, root, stem, leaf, ovary, and fruit peels from the two parental lines. As shown in **Figure 5A**, the expression level of the stigma decreases as it grows from immature to blossom stage in both lines. However, regardless of the stigma developmental stages, the green stigma bitter gourd still showed a significant high expression level compared with the yellow stigma gourd. In addition, *McAPRR2* shows a higher expression level in S051 than in S093 across all organ tissues (**Figure 5B**). The expression level of *McAPRR2* was relatively low in the petals of both female and male flowers. Combined with the results of fine mapping and expression analysis, it is inferred that *McAPRR2* is the best candidate for *McSTC1 locus*. The interrupting expression of *McAPRR2* may result in less accumulation of chlorophyll that result in yellow stigma color in bitter gourd.

### 3.6 Phylogenetic analysis

It has been revealed that the APRR2 orthologs in the cucurbit plants are associated with plastid number and the chloroplast development (Liu et al., 2016; Jiao et al., 2017; Oren et al., 2019). By conducting a genome-wide survey of the *APRR2* gene in the genome of bitter gourd, two *APRR2* genes, *Mc06g1638/Moc06g35570* (this study) and *Mc10g1288/Moc10g28360*, are identified in both Dali-11 v1 and OHB3-1 v2 reference genome assembly. Thus, to further understand the functional relationship of *APRR2* genes within other cucurbit crops, we performed a genome-wide synteny search of the two bitter gourd *McAPRR2* genes with eight crops from Cucurbitaceae family, including cucumber (*Cucumis sativus*), melon (*Cucumis melo*), Watermelon (*Citrullus lanatus*), Sponge gourd (*Luffa cylindrica*), Bottle gourd (*Lagenaria siceraria*), Wax gourd (*Benincasa hispida*), *Cucurbita moschata*, and *Cucurbita maxima* (Yu et al., 2022). Similar to bitter gourd, most investigated cucurbit crops here harbored two copies of *APRR2* genes, with the exception of cucumber and *Cucurbita* (*Cucurbita moschata* and *Cucurbita maxima*), whose genomes encode a total of three and seven *APRR2* genes, respectively (**Table S5**). The two copies of *APRR2* gene in majority of cucurbit crops and the extended *APRR2* genes in *Cucurbita* are probably the result of the first step of whole-genome duplication (WGD) event at the origin of the Cucurbitaceae family and tandem-duplications at Cucurbiteae tribe, respectively (Sun et al., 2017; Guo et al., 2020; Ma et al., 2022).

**Figure 6:**
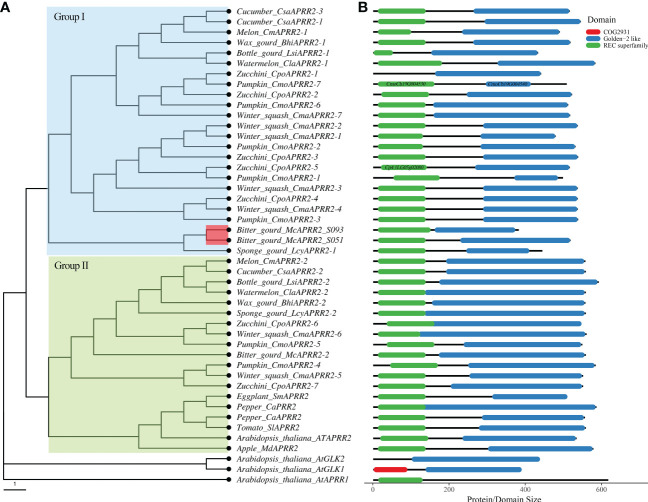
Phylogenetic analysis of *APRR2* from Cucurbitaceae family and other plant species. **(A)** The neighbor-joining tree of *APRR2*.**(B)** The domain demonstration of *APRR2*. Genes information is provided in **Table S5**.

To dissect the evolutionary relationship of *APRR2* genes, we further performed a phylogenetic analysis using neighbor-joining method for the *APRR2* genes from Cucurbitaceae family and other plant species including Arabidopsis, Tomato, Apple, and Pepper (**Figure 6A**). It clearly shows that the Arabidopsis *GLK1/2* and *AtAPRR1* clade is distinct from the *APRR2* clade of all other plants. All of the *APRR2* contained two typical domains: Rec super family and Golden-2 like domain (PLN03162), which corresponded to the myb SHAQKYF domain according to InterproScan and Conserved Domian Databased (CDD) (**Figure 6B**). In addition, the *APRR2* genes from Cucurbits crops were clustered into two groups according to their similarities in amino acid sequence. The genes in Group I and Group II share partial and complete Golden-2 like domain, respectively. The Group I genes include those APRR2 genes that have been shown to correlate to the white or pale green fruit skin such as cucumber *CsAPRR2-3*, melon *CmAPRR2-1*, watermelon *ClaAPRR2-1*, and wax gourd *BhAPRR2-1* (**Figure 6A**; Liu et al., 2016; Oren et al., 2019; Ma et al., 2021). In addition, the Zucchini *CpoAPRR2-1* and *CpoAPRR2-2* were found associated with light green stem color (Zhu et al., 2022). The bitter gourd *McAPRR2* reside in Group I as well (**Figure 6A**). In particular, the two adjacent genes *CmoCh19G004530* and *CmoCh19G004540* in Pumpkin reference genome (*Cucurbita moschata*) were annotated to have the REC and Golden-2 like domain, respectively. We further re-annotated the two genes using FGENESH which indicated that they belong to a single APRR2 gene. Thus, we designated them as *CmoAPRR2-7* accordingly (**Table S5**; **Figure 6B**).

## 4 Discussion

### 4.1 Mapping of stigma color gene in bitter gourd

In the present study, we conducted BSA-seq of the *Stigma color (McSTC1)* locus in bitter gourd inbred lines S051 and S093, and delimited it into a 13.87 kb interval at MC06 that contains only one gene, *Mc06g01638* (*McAPRR2*) (**Figure 3**). The gene expression and allelic diversity analyses supported *McAPRR2* as the candidate gene for *McSTC1* (**Figures 4**, **5**). *McAPRR2* is a homolog of Arabidopsis *AtAPRR2* (*AT4G18020*) that is a plant specific transcription factor belonging to the Pseudo-Response Regulator (PRR) family originated from Authentic Response Regulator (ARRs) (Makino et al., 2000). We further cloned the CDS of *McAPRR2* in the two parental lines *via* sanger sequencing; combined with BSA-seq results, we confirmed the two non-synonymous SNP variants (SNP.A23824615G and SNP.A23825627G) and two InDels (39 bp and 15 bp insertion in S093) (**Figure 4A**). The 15 bp insertion were designed as InDel markers to genotype 45 bitter gourd inbred lines mainly collected from Southern Asia countries; the present-absent of 15 bp sequence showed a completely agreement with their stigma color with all yellow stigma inbred lines harbor the 15 bp insertion (**Figure 4B**; **Table S6**). The two non-synonymous SNP variants were examined through Sanger sequencing: the SNP.A23825627G is consistent with expected phenotypes while SNP.A23824615G showed discrepancies (**Table S6**). Although the examined collected inbred lines are not abundant, it still suggests a greatly potential of applying the 15 bp InDel marker and SNP markers for bitter gourd MAS breeding.

An early study conducted using an F_2_ population derived from a cross between bitter gourd inbred lines “Taiwan White” and “CBM12” also revealed a single dominant locus for stigma color *StCol* which was mapped to LG1 linked with AFLP markers E19M57h and E18M53b; due to the limitation of marker information, its physical position is unknown (Kole et al., 2012). In addition, Kole et al. (2012) reported the independently inherited nature of *StCol* that is not associated with other investigated fruit traits including fruit skin color, fruit surface structure, and seed color. In contrast to *McSTC1*, *StCol* displays a predominance of yellow stigma over green stigma, indicating the existence of other regulatory mechanisms governing the stigma color in bitter gourd. An additional genome-wide association investigation using a widely collected large population may be able to enhance our knowledge of the genetic variability of bitter gourd stigma color (Cui et al., 2022).

### 4.2 *McAPRR2* is associated with chlorophyll accumulation in stigma of bitter gourd

In Cucurbit crops, green and yellow stigma are two colors have been observed (Lv et al., 2022b). The green color of stigmas is likely caused by accumulation of chlorophyll, the same pigment that gives plants leaves and fruits their characteristic green appearance. The genetic study for melon (*Cucumis melo*) stigma color showed that green stigma is dominant over the yellow one; transcriptomic analysis proposed that the yellow color is probably due to the break-down of chloroplast structure interfering with chlorophyll biosynthesis; in contrast, the green stigmas are a functional accumulation of chlorophyll (Lv et al., 2022a; Lv et al., 2022b). This might also be true for the green/yellow stigma of bitter gourd. In the present study, we showed that *McAPRR2* is the best candidate gene for *McSTC1* locus; particularly the loss-of function allele caused by 15 bp-insertion in S093 resulted in yellow stigma. *McAPRR2* is a homolog of *Arabidopsis pseudo-response regulator 2* gene (*APRR2*). A typical PRR2 possesses a receiver-like domain (RLD) and a Golden-2 like (GLK) domain that contains a Myb-like DNA binding domain (Hosoda et al., 2002; Chen et al., 2016). The GLKs transcription factors (TFs) belong to a GARP superfamily TF whose functions are important for the expression of nuclear photosynthesis-related genes and for chloroplast development in both leaves and fruits (Riechmann et al., 2000; Hosoda et al., 2002; Waters et al., 2008; Nguyen et al., 2014). The *APRR2* orthologs have been shown to regulate chlorophyll content in a wide range of crops from Solanaceae and Cucurbitaceae including tomato, pepper, eggplants, cucumber, melon, watermelon, wax gourd, and Zucchini (Pan et al., 2013; Liu et al., 2016; Oren et al., 2019; Jeong et al., 2020; Ma et al., 2021; Arrones et al., 2022; Zhu et al., 2022; Fang et al., 2023). These genes are found to associate with increased plastid numbers and chlorophyll accumulation of fruit colors in immature fruit peels, resulted in white or light green. The cucumber *CsAPRR2* served as TF contributing to the chloroplast development was reported through the interaction with *Class-I KNOTTED 1-like homeobox* (*KNOX*) genes *TKN4* and *TKN2* (Jiao et al., 2017). Particularly, the *KNOX* genes play an important role in plant hormone cytokine (CTK) network for its role in regulating the development and activity of chloroplasts (Cortleven and Schmülling, 2015). It is reasonable to proposed that loss of function of *McAPRR2* allele in S093 may impair its interaction ability with downstream genes for its regulation roles in the chloroplast development in stigma tissues, resulting in a recessive yellow coloration. In addition, the relative expression level of *McAPRR2* in S051 with green stigma is significantly higher than that in S093 with yellow stigma throughout stigma developmental stages, as well as majority of the organ tissue (except for petals). According to the result from BSA-seq, a number of 14 variants at ~2 kb upstream of *McAPRR2* were found (highlight in **Table S3**, **L2650-2663**). Whether they are responsible for the differential expression between the two parental lines requires further analysis. In addition, the fruits of S093 bear lighter green fruit skin compared with that of S051 line (**Figure S4**); nevertheless, additional work is required to determine whether the *McAPRR2* gene is also responsible for the fruit skin color of bitter gourd.

### 4.3 The Cucurbits *APRR2* genes underwent whole genome duplication event

Throughout the plant evolutionary history, the Cucurbitaceae plants experienced at least four whole-genome duplication (WGD) events (Guo et al., 2020). The first large-scale duplication event is the cucurbit-common tetraploidization (CCT) occurred at the origin of Cucurbit crops (115-130 Mya) (Wang et al., 2018; Guo et al., 2020; Ma et al., 2022). The CCT event likely contribute to the presence of two copies of *APRR2* in bitter gourd and other cucurbits crops, as disclosed by a genome-wide syntenic search for Cucurbitaceae family. Additionally, a cluster of *APRR2* genes from *Cucurbita* were showed in the phylogenetic tree (*CmoCh02G016320-350* and *CmaCh02G015890-930*), that likely evolved in response to a lineage-specific recent WGD event (Sun et al., 2017; Ma et al., 2022).

The *APRR2* genes from cucurbit crops fall into two groups (I and II) accordingly and both groups have conserved REC superfamily and GLK2 superfamily domains. The genes in Group I and Group II are mainly distinguished by the presence of partial and complete Golden-2 like (GLK) domain, respectively (**Figure 6B**). The Group I clade includes the *CsAPRR2* in cucumber, *CmAPRR2* in melon, *ClaAPRR2* in watermelon, *BhAPRR2* in wax gourd which have been shown to correlate with white or light green fruit skin and *CpoAPRR2* in Zucchini is associated with stem color (**Table S5**, **Figure 6A**; Liu et al., 2016; Oren et al., 2019; Ma et al., 2021; Zhu et al., 2022). *McAPRR2*, the candidate gene in regulating green stigma color of bitter gourd, is clustered with the genes in Group I as well (**Figure 6A**). It is reasonable to speculate that *McAPRR2* may also play a role in chloroplast development for fruit skin color, since the line S093 bears lighter green fruit skin of bitter gourd compared with line S051(**Figure S4**). A further investigation of the fruit skin color using the same population might aid in dissolving the involvement of *McAPRR2*. In addition, Further studies are needed to determine if the two *APRR2* copies act redundantly or specifically in coordinating the control of chloroplast development throughout all fruit tissues and developmental stages.

## Data availability statement

The datasets presented in this study can be found in online repositories. The names of the repository/repositories and accession number(s) can be found in the article/**Supplementary material**.

## Author contributions

JYZ and JZ performed majority of the experiments. YW participated the data analysis. KH and JC conceive and supervised the study. YW wrote the manuscript with JYZ. All authors contributed to the article and approved the submitted version.
