# Supplementary material for: Map-based cloning of the APRR2 gene controlling green stigma in bitter gourd (Momordica charantia)
Source: Front Plant Sci. 2023 May 10;14:1128926. doi: 10.3389/fpls.2023.1128926 (PMC10208069; doi:10.3389/fpls.2023.1128926)
Supplement: Supplementary file 1 [file Presentation_1.pdf]

Supplemental Material

1 Supplementary Figures

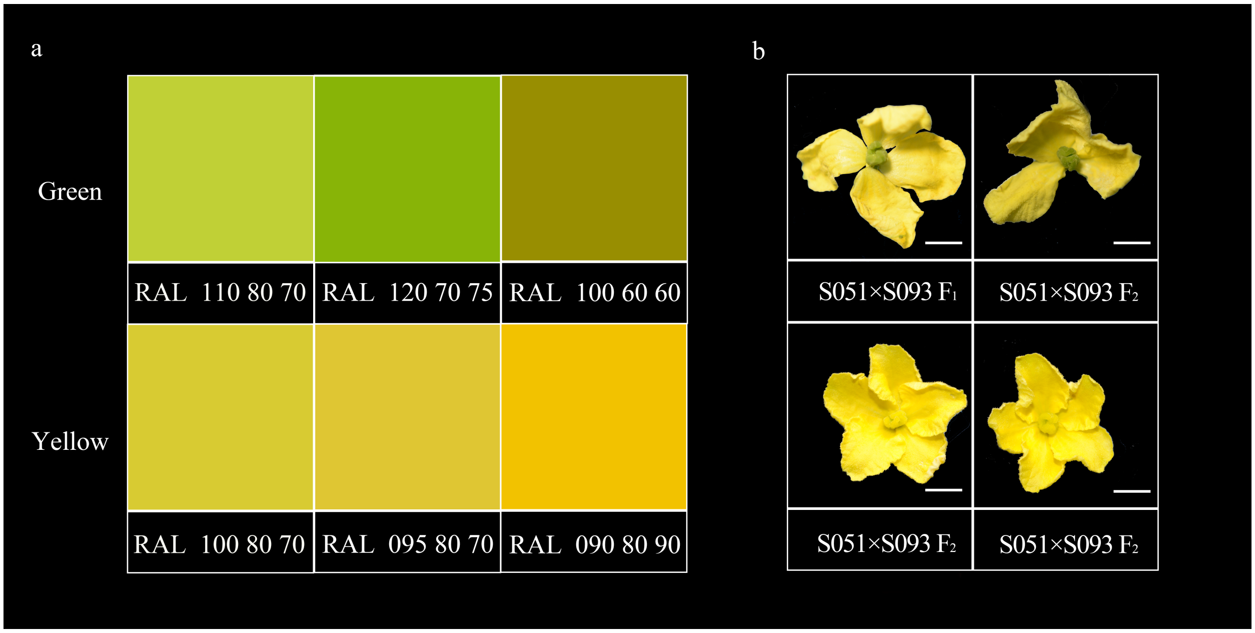

Supplementary Figure 1. a) The classified standard of stigma color based on RAL D9 DESIGN CYMPHONY OF COLOURS color card. **b)** The female flower photos of S051×S093 F_1 and F_2 individuals. Bars in female flowers represent 1 cm.

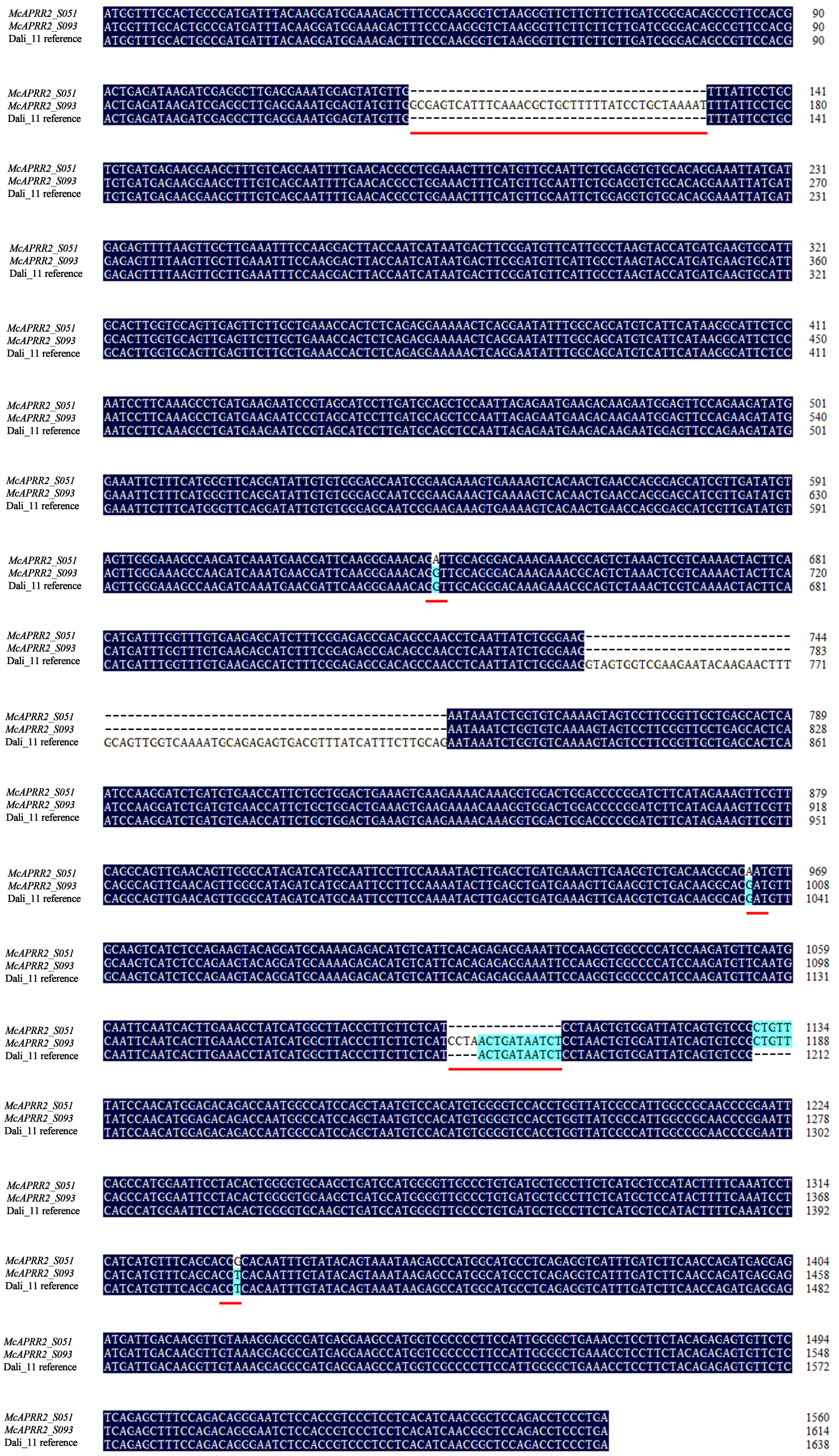

Supplementary Figure 2. CDS alignment among parental lines (S051 and S093) and Dali-11 reference. Red line indicates the variants between S051 and S093. Gene sequences of McAPRR2 from S051 and S093 were deposited in NCBI with GenBank accession numbers of OP972606 and OP972607, respectively.

```
McAPRR2_S051   MVCTADDLQGWKDFPKGLRVLLLDRDSRSTTEIRSRLEEMEYV------------VYSCCDEKEALSAILNTPGNFHVA   67
McAPRR2_S093   MVCTADDLQGWKDFPKGLRVLLLDRDSRSTTEIRSRLEEMEYVGESFQTLLFILLKFYSCCDEKEALSAILNTPGNFHVA   80

McAPRR2_S051   ILEVCTGNYDESFKLLEISKDLPIIMTSDVHCLSTMMKCIALGAVEFLLKPLSEEKLRNIWQHVIHKAFSNPSKPDEESV   147
McAPRR2_S093   ILEVCTGNYDESFKLLEISKDLPIIMTSDVHCLSTMMKCIALGAVEFLLKPLSEEKLRNIWQHVIHKAFSNPSKPDEESV   160

McAPRR2_S051   ASLMQLQLENEDKNGVPEDMEILSWVQDIVWEQSEESEKSQLNQGASLICSWESQDQMNDSRETDCRDKETQSKLVKTTS   227
McAPRR2_S093   ASLMQLQLENEDKNGVPEDMEILSWVQDIVWEQSEESEKSQLNQGASLICSWESQDQMNDSRETGCRDKETQSKLVKTTS   240

McAPRR2_S051   HDLVCEEHLSESDSQPQLSGKNKSGVKSSPSVAEHSIQGSDVNHSAGLKVKKTKVDWTPDLHRKFVQAVEQLGIDHAIPS   307
McAPRR2_S093   HDLVCEEHLSESDSQPQLSGKNKSGVKSSPSVAEHSIQGSDVNHSAGLKVKKTKVDWTPDLHRKFVQAVEQLGIDHAIPS   320

McAPRR2_S051   KILELMKVEGLTRHNVASHLQKYRMQKRHVIHREEIPRWPHPRCSMQFNHLKPIMAYPSSHPNCGLSVSAVYPTWRQTNG   387
McAPRR2_S093   KILELMKVEGLTRHDVASHLQKYRMQKRHVIHREEIPRWPHPRCSMQFNHLKPIMAYPSSHPN*                  383

McAPRR2_S051   HPANVHMWGPPGYRHWPQPGIQPWNSYTGVQADAWGCPVMLPSHAPYFSNPHHVSAPHNLYTVNKSHGMPQRSFDLQPDE   467
McAPRR2_S093

McAPRR2_S051   EMIDKVVKEAMRKPWSPLPLGLKPPSTESVLSELSRQGISTVPPHINGSRPP*                              519
McAPRR2_S093
```

Supplementary Figure 3. Amino acid sequence alignment between parents (S051 and S093). * represents the terminator.

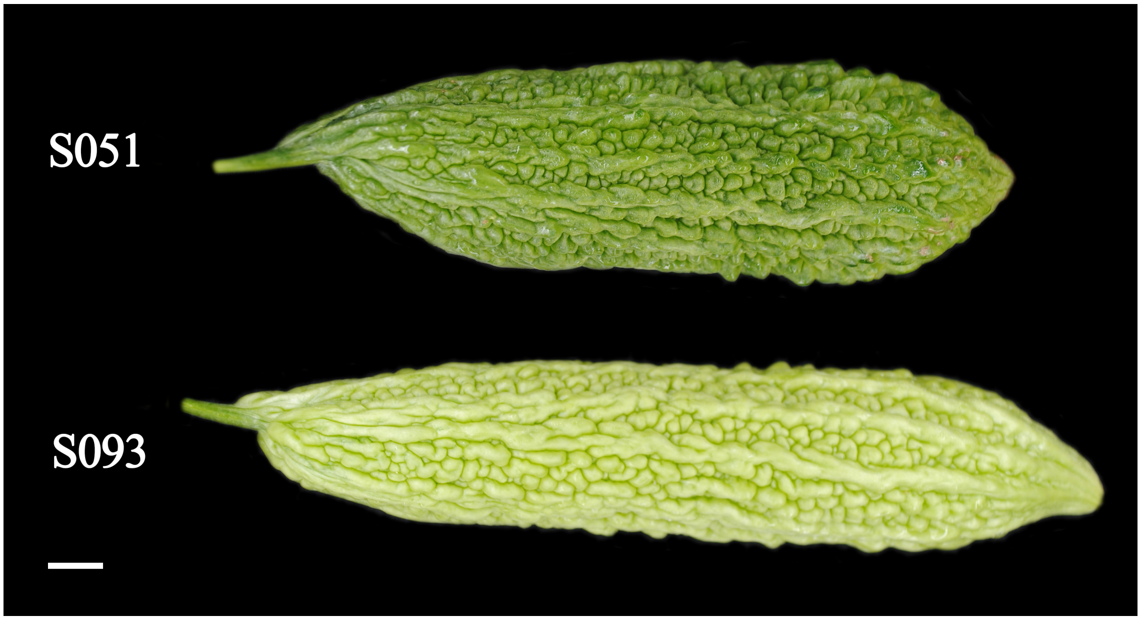

Supplementary Figure 4. The fruits photos of parental lines S051 and S093. Bar represents 1 cm.
